# RNA-binding proteins that lack canonical RNA-binding domains are rarely sequence-specific

**DOI:** 10.1038/s41598-023-32245-9

**Published:** 2023-03-31

**Authors:** Debashish Ray, Kaitlin U. Laverty, Arttu Jolma, Kate Nie, Reuben Samson, Sara E. Pour, Cyrus L. Tam, Niklas von Krosigk, Syed Nabeel-Shah, Mihai Albu, Hong Zheng, Gabrielle Perron, Hyunmin Lee, Hamed Najafabadi, Benjamin Blencowe, Jack Greenblatt, Quaid Morris, Timothy R. Hughes

**Affiliations:** 1 Donnelly Centre, University of Toronto, Toronto, ON M5S 3E1 Canada; 2 Department of Molecular Genetics, University of Toronto, Toronto, ON M5S 1A8 Canada; 3 Department of Human Genetics, McGill University, Montréal, QC H3A 0C7 Canada; 4 McGill Genome Centre, Montréal, QC H3A 0G1 Canada; 5 Computational and Systems Biology Program, Memorial Sloan Kettering Cancer Center, New York, NY USA; 6 Tri-Institutional Training Program in Computational Biology and Medicine, Weill Cornell Medicine, New York, NY USA

**Keywords:** Computational models, RNA-binding proteins

## Abstract

Thousands of RNA-binding proteins (RBPs) crosslink to cellular mRNA. Among these are numerous unconventional RBPs (ucRBPs)—proteins that associate with RNA but lack known RNA-binding domains (RBDs). The vast majority of ucRBPs have uncharacterized RNA-binding specificities. We analyzed 492 human ucRBPs for intrinsic RNA-binding in vitro and identified 23 that bind specific RNA sequences. Most (17/23), including 8 ribosomal proteins, were previously associated with RNA-related function. We identified the RBDs responsible for sequence-specific RNA-binding for several of these 23 ucRBPs and surveyed whether corresponding domains from homologous proteins also display RNA sequence specificity. CCHC-zf domains from seven human proteins recognized specific RNA motifs, indicating that this is a major class of RBD. For Nudix, HABP4, TPR, RanBP2-zf, and L7Ae domains, however, only isolated members or closely related homologs yielded motifs, consistent with RNA-binding as a derived function. The lack of sequence specificity for most ucRBPs is striking, and we suggest that many may function analogously to chromatin factors, which often crosslink efficiently to cellular DNA, presumably via indirect recruitment. Finally, we show that ucRBPs tend to be highly abundant proteins and suggest their identification in RNA interactome capture studies could also result from weak nonspecific interactions with RNA.

## Introduction

RNA-binding proteins (RBPs) control diverse RNA-related processes, ranging from RNA splicing to anti-viral defense, significantly impacting cellular and physiological function^1–7^. The human genome encodes over 400 proteins that contain well-studied RNA-binding domains (RBDs)^8^, but genome-wide RNA interactome capture assays using mass spectrometry have collectively cataloged thousands of proteins that crosslink to mRNA and non-coding RNA^9–11^. Many of these proteins have no previously reported function in RNA-binding, regulation, or metabolism. These new “unconventional” RBPs (ucRBPs)^12,13^—also referred to as enigmRBPs^14^, “non-canonical”, “non-classical”, and “non-professional” RBPs^15^—lack canonical RBDs and represent a wealth of potential new factors in RNA biology. Despite their prevalence, it is unclear how many ucRBPs recognize specific RNA sequences and structures. Some well-known ucRBPs are clearly sequence-specific (e.g. CFI(m)/NUDT21^16^, Vts1p^17^, ZRANB2^18^, and others listed below), but more than a decade after the initial mass spectrometry studies, most remain uncharacterized in this regard.

The existence of so many ucRBPs also raises the question of how many sequence-specific RBDs remain to be discovered. Relative to transcription factor DNA-binding domains, which number well over 100 among eukaryotes^19^, there are relatively few types of classical sequence-specific RBDs, with most of the literature focused on RRM, KH, CCCH zinc finger (CCCH-zf), and Pumilio domains^8,20–23^. Many more types of protein domains are associated with RNA metabolism^24^, and thus presumably have affinity for RNA, but few have reported sequence specificity. A handful of domain types (e.g. NHL)^25,26^ appear to have evolved RNA-binding sequence specificity in some phylogenetic branches^27^, presumably derived from predecessors with other biochemical functions. Proteins that form ribonucleoprotein complexes, such as the ribosome, spliceosome, and telomerase, among others, represent a special case. These proteins are associated with a single major substrate, but there is evidence that many perform “moonlighting” functions beyond their well-established roles^28^.

Here, we surveyed a panel of 492 ucRBPs to determine their intrinsic RNA sequence preferences, subsequently localizing several RBDs and exploring the sequence specificity of their homologs. We anticipated that many new sequence-specific RBPs and their associated RBDs would emerge but, instead, very few of either were identified beyond those that were already known. This outcome suggests that although some ucRBPs may have roles in RNA metabolism, they do not rely on RNA sequence specificity. Alternatively, there are other explanations for their detection in RNA interactome capture experiments; we suggest a few below.

## Results

### Analysis of 492 ucRBPs using RNAcompete

Initially, we curated a set of 525 ucRBPs from two initial studies that identified RBPs crosslinked to mRNA at a genome-wide level^9,10^. Starting from a merged list of approximately 1100 putative RBPs, we removed any that contained RRM, KH, CCCH-zf, or Pumilio domains. Additionally, we removed any that were greater than 600 amino acids long, as large proteins are less compatible with expression and purification from *E. coli*. Several of the remaining 525 ucRBPs were already known or have since been found to recognize specific RNA-binding motifs (NUDT21^12,16^, SERBP1^29^, CNBP^12,30^, NHP2L1^31^, ZRANB2^18^, and SLBP^32^), and these served as internal controls. Others are known to interact with RNA but have more limited information on sequence specificity (e.g. IFIT2^33^ NUDT16L1^34^, RPL22^35^, and others below), but we did not exhaustively survey the literature on all 525 proteins in advance. Furthermore, the experiments were conducted in parallel with hundreds of additional proteins containing conventional RBDs (from Sasse et al., to be described elsewhere, and other collaborative studies).

From our list of 525 ucRBPs, we successfully expressed and purified 492 full-length GST fusion proteins and analyzed them using RNAcompete^36^. Briefly, in RNAcompete experiments, a purified GST-tagged RBP selects RNA sequences from a designed (non-randomized) RNA pool. This pool is generated from a custom Agilent 244 K microarray consisting of 241,399 30–41 base RNAs. Following the GST pulldown, RNAs bound to the RBP are isolated, labeled with fluorescent Cy3 or Cy5 dyes, and hybridized to another custom 244 K Agilent microarray. Afterwards, the fluorescent intensities of individual microarray spots are quantified and used to estimate the level of RNA-binding by RBPs to specific RNA pool sequences. Computational analysis of RNAcompete microarray data calculates Z-score values for an RBP of interest to all RNA 7-mer sequences, representing the preference of an RBP to individual RNA 7-mers (i.e. relative RNA-binding affinity). The 7-mers with the highest Z-scores, which represent 7-mers that are bound with the highest affinity, are then aligned, and used to generate RNA-binding motifs. A design feature of the RNA pool is that RNA sequences in the starting pool can be split computationally into two sets, “Set A” and “Set B”, which have a nearly equal distribution of 7-mers. We use this feature to produce an internal reproducibility control by comparing 7-mer scores and motifs calculated separately for each set.

**Figure 1:**
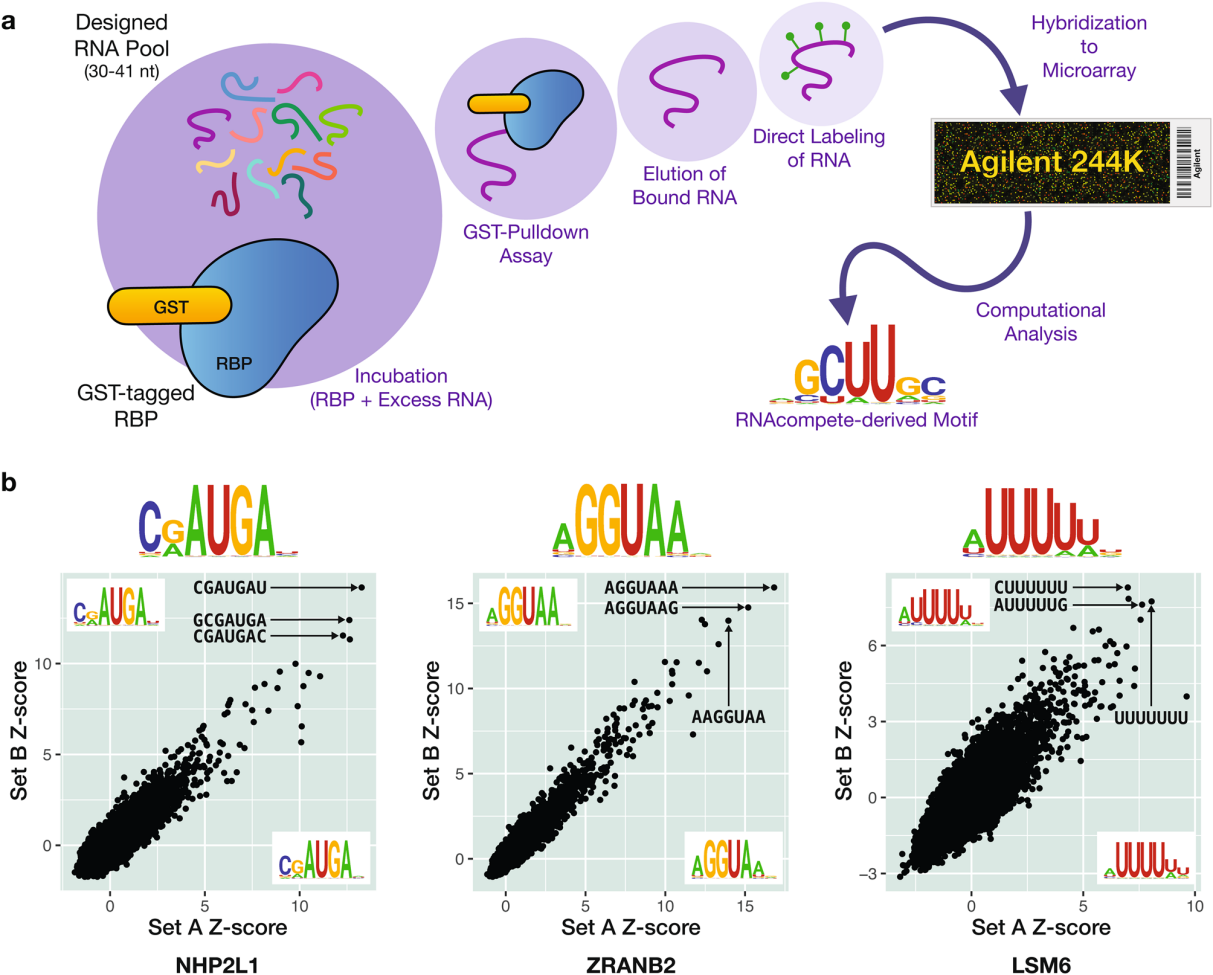
Schematic for RNAcompete assays and sample experimental data. (**a**) A GST-tagged RBP (RBP is blue shape, GST-tag is yellow oval) is incubated with a 75-fold excess of a non-random, custom designed RNA pool (multicolored lines). RNA selectively bound to an RBP during a GST-pulldown assay is eluted, directly labeled with either Cy3 or Cy5 (green circles) and hybridized to a custom Agilent 244 K microarray. Microarray data is analyzed computationally to generate RNA-binding motifs, represented as logos. (**b**) RNAcompete sample data for several classes of ucRBPs—NH2PL1 (“Ribosomal proteins” ucRBP class), ZRANB2 (previously characterized ucRBP class), and LSM6 (“Other” ucRBP class)—represented in Fig. 2. Scatterplots show correlation between 7-mer Z-scores for Set A and Set B sequences. RNAcompete logos derived from the top 10 7-mers from Set A sequences (bottom right corner of scatterplot), Set B sequences (top left corner of scatterplot), and the combined Set A and Set B sequences (top of scatterplot) are indicated. The top three 7-mer sequences are shown in the plots. Figures 1, 2, 3, 4, 5 and 6 were created using Adobe Illustrator version 25.4.1—motifs were made using R (version 4.1.3) with the ggseqlogo package (version 0.1) and scatter plots/histograms were made in R (version 4.1.3) with the ggplot2 package (version 3.3.5, https://ggplot2.tidyverse.org).

A schematic and example data from this study are shown in Fig. 1, and details of all RNAcompete experiments, including ucRBP protein sequences, are provided in Supplementary Table S1. We cloned, purified, and analyzed the ucRBPs in batches that included many proteins from other projects done in the laboratory in parallel. These concurrent experiments served as process controls and as direct comparisons for general outcome of the study.

### A small proportion of ucRBPs display clear sequence specificity

RNAcompete generates data that is conceptually straightforward. A successful experiment is typically characterized by a subset of related 7-mers yielding relatively high Z-scores and clear RNA motifs that are shared between Set A and Set B (as in Fig. 1B) (Z > 5 would correspond to Bonferroni-corrected P < 0.005, assuming a normal distribution). In concurrent experiments with conventional RBPs (containing mainly RRM, KH, and CCCH-zf domains from diverse eukaryotes), high-scoring 7-mers and motifs for sequence-specific RBPs were readily identified 57% of the time, illustrating that the assay is robust. We note that some level of failure is expected, as almost all of these were previously uncharacterized proteins, and not all of them may be *bona fide* RBPs.

In our initial manual analysis of the data, ucRBPs overall displayed a much lower success rate than conventional RBPs. We obtained previously reported motifs for four of the five internal ucRBP controls (NUDT21, SERBP1, CNBP, and ZRANB2; SLBP is addressed below). Overall, only 63 of the 492 displayed any indication of sequence specificity, however, and many had low Z-scores and/or poor correlation between the A and B sets. All 63 were replicated, and most were judged to be not reproducible. To ensure unbiased assessments for the ucRBP (and other) RNAcompete experiments, we developed an automated classifier that combined a panel of RNAcompete experimental outcomes into a (pass/fail/uncertain) scoring system (Supplementary Fig. S1, Supplementary Table S3). This system was trained on the hundreds of concurrent experiments performed with conventional RBPs (i.e. uncharacterized proteins with RRM, KH, and CCCH domains). Classifier assignments for the ucRBP experiments were nearly identical to manual assignments, with only 34/558 (6.1%) experiments (492 RBPs, 66 replicates, including three RBPs run in triplicate) scoring as “successful”. The system flagged an additional 17/558 (3.0%) experiments as “uncertain”, of which we “passed” eight upon manual inspection (see “Methods”). Among all 63 ucRBPs with replicates, 49 were assigned the same class in both replicates, indicating a low error rate for our coupled experimental/computational system; the remainder were largely borderline cases (slightly above or below the corresponding threshold) and were resolved manually.

**Figure 2:**
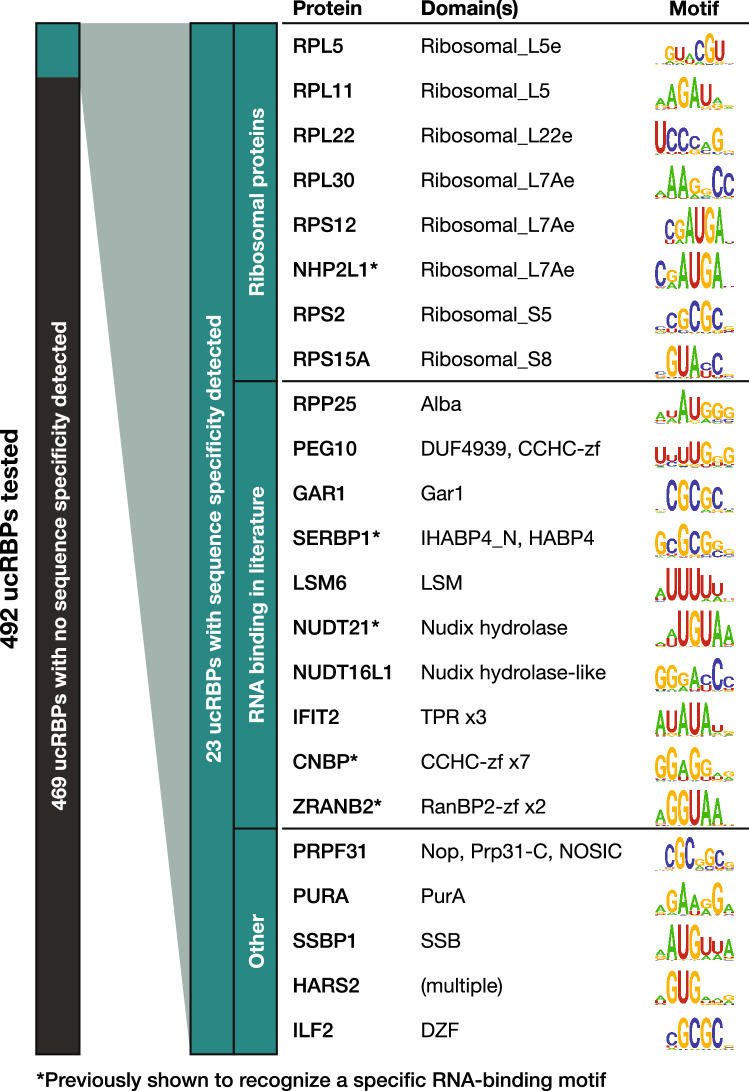
Large-scale analysis of intrinsic ucRBP RNA-binding specificity. A total of 23 sequence-specific ucRBPs were discovered among a panel of 492 and grouped into three classes: “Ribosomal proteins”, those associated with “RNA-binding in the literature”, and “Other,” which likely represent new sequence-specific ucRBPs. ucRBP gene names, corresponding protein domains, and RNAcompete-derived motif logos are presented.

In total, after merging replicates, we obtained sequence-specific RNA-binding motifs for 23 unique ucRBPs (Fig. 2). We grouped these into three classes. The first class (eight proteins) is comprised of ribosomal proteins, or proteins with domains found in ribosomal proteins. The second class (ten proteins) corresponds to non-ribosomal proteins that are known to bind RNA, including instances with limited information on sequence specificity (i.e. the RNAcompete motifs represent new consensus sequences)^12,16,29,30,37^. For example, we identified putative consensus sequences for IFIT2 which has only been shown to bind a small number of A/U-rich oligos^33^, and LSM6 which is a structural component in LSM complexes but has limited contact with RNA and has not been shown to bind specific RNA motifs^38^. The third class (five proteins) corresponds to ucRBPs that, to our knowledge, have not been previously shown to possess RNA-binding activity. Thus, a key outcome of this study is the identification of several novel *bona fide* sequence-specific RBPs.

### Dissection and exploration of potential new RBDs

**Figure 3:**
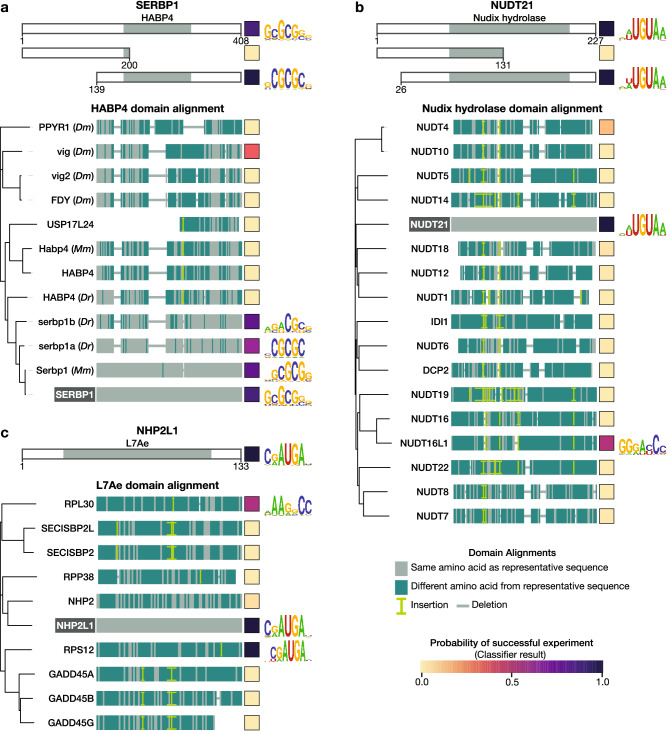
RNA-binding specificity of putative ucRBDs (Part I). Representative ucRBPs identified in this study for selected ucRBDs—(**a**) HABP4, (**b**) Nudix hydrolase, and (**c**) L7Ae—are shown at the top. Below these are depictions of deletion constructs used to identify corresponding ucRBDs with sequence-specific RNA-binding. ucRBPs listed below the ucRBD “domain alignment” heading depict ucRBD-only constructs analyzed by RNAcompete. Amino acid differences between the representative and test ucRBDs, as analyzed by COBALT^83^, are indicated. Clustal Omega^82^ was used to generate phylogenetic trees; visualizations were created with iTOL^88^. Square boxes to the right of each construct display the classifier score indicating the probability of a successful RNAcompete experiment. Logos are provided for ucRBPs/ucRBDs with sequence-specific RNA-binding motifs. *Mm*, *Mus musculus*; *Dr*, *Danio rerio*; *Dm*, *Drosophila melanogaster*.

**Figure 4:**
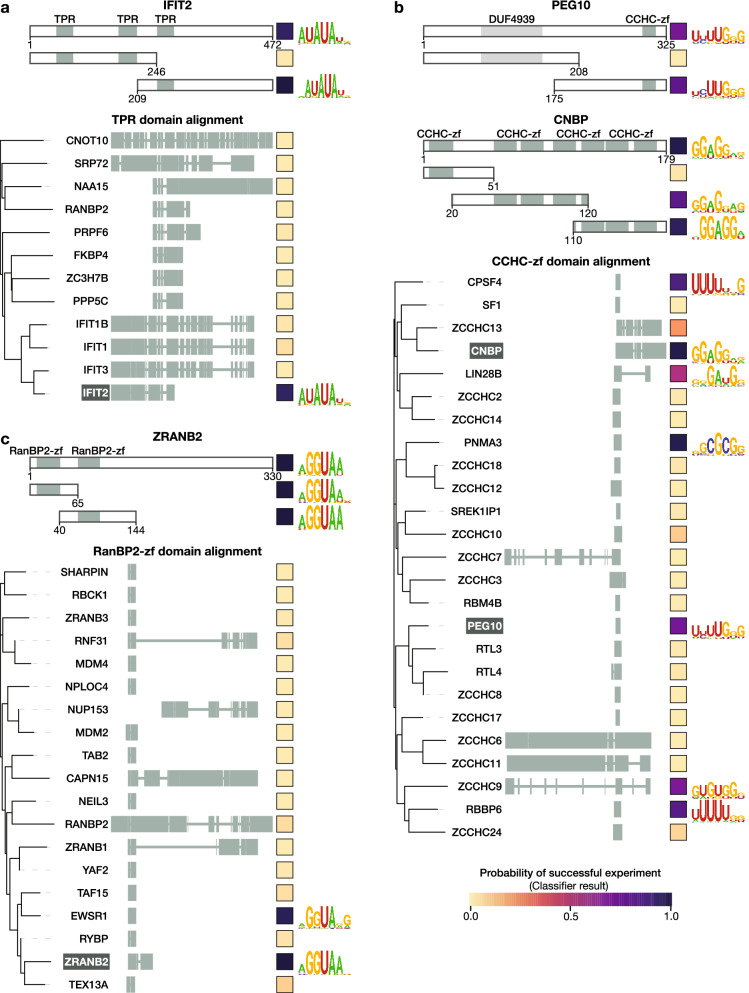
RNA-binding specificity of putative ucRBDs (Part II). Representative ucRBPs identified in this study for selected ucRBDs—(**a**) TPR, (**b**) CCHC-zf, and (**c**) RanBP2-zf—are shown at the top. Below these are depictions of deletion constructs used to identify corresponding ucRBDs with sequence-specific RNA-binding. ucRBPs listed below the ucRBD “domain alignment” heading depict ucRBD only constructs analyzed by RNAcompete. Clustal Omega^82^ was used to generate phylogenetic trees; visualizations were created with iTOL^88^. Square boxes to the right of each construct display the classifier score indicating the probability of a successful RNAcompete experiment. Logos are provided for ucRBPs/ucRBDs with sequence-specific RNA-binding motifs.

The ucRBPs yielding motifs often contained annotated protein domains that are associated with RNA-binding, but the RNA sequence specificity of these domains, and their prevalence in RNA-binding, has not been extensively studied (Fig. 2). We selected a panel of unconventional RNA-binding domain (ucRBD) candidates, generated deletion constructs containing putative ucRBDs, and analyzed their RNA-binding specificities using RNAcompete. This panel of candidates was comprised of HABP4 (from SERBP1), Nudix hydrolase (from NUDT21), L7Ae (from NHP2L1), RanBP2-zf (from ZRANB2), CCHC-zf (from PEG10 and CNBP), and TPR (from IFIT2). Strikingly, numerous ucRBD(s) deletion constructs contained sequence-specific RNA-binding activity nearly identical to their corresponding full-length ucRBPs (Figs. 3, 4). These results are consistent with the literature for several of the well-characterized ucRBPs that were selected—CNBP, SERBP1, NHP2L1, NUDT21, and ZRANB2^16,18,30,39,31^—and novel for the less-well studied ucRBPs — IFIT2 (TPR domain) and PEG10 (CCHC-zf domain).

We then expanded the scope of this analysis by assessing whether homologs of these ucRBDs also bind RNA in a sequence-specific manner (Fig. 3). Here, we generated a panel of 89 proteins comprised of the six types of ucRBDs examined above—HABP4 (11), Nudix hydrolase (16), RanBP2-zf (18), CCHC-zf (24), L7Ae (9), and TPR (11) domains—and surveyed their RNA-binding specificities, using RNAcompete. The selected proteins encompassed all human CCHC-zf, L7Ae, HABP4, and RanBP2-zf domain-containing proteins that had not been previously analyzed by RNAcompete. We randomly selected subsets of Nudix hydrolase and TPR domain-containing proteins (with similarity to IFIT2), and a selection of HABP4 domain-containing proteins across metazoans. For the human HABP4 domain, only closely related orthologs from mouse (Serbp1; 98% identity) and zebrafish (serbp1a; 63% identity, and serbp1b; 72% identity) yielded motifs similar to human SERBP1, but more dissimilar HABP4 domains (less than 50% identity) did not (Fig. 3). In another example, the RanBP2-zf domain from EWSR1, which has 59% identity to the first RanBP2-zf domain from ZRANB2, bound a very similar RNA motif, but none of the other RanPB2-zf domains yielded motifs. None of the TPR domain constructs besides IFIT2 yielded motifs. In contrast, three very different L7Ae domains, with protein identity as low as 12%, displayed RNA sequence specificity, as did two very different Nudix hydrolase domains from previously studied RBPs (NUDT21 and NUDT16L1). These examples are consistent with evolution of RNA-binding through co-option of a domain that would typically have another function. Interestingly, for L7Ae and Nudix hydrolase, the derivation of sequence-specific RNA-binding function has occurred more than once in the lineage leading to human.

A particularly striking outcome of this analysis is that seven of the 25 human proteins with CCHC-zf ucRBDs yielded a clear primary sequence motif (Fig. 4). CCHC-zf proteins have been associated with RNA-related function and RNA-binding^40,41^, but the CCHC-zf domain is not generally considered to be among canonical sequence-specific RBD families (e.g. RRM, KH, CCCH-zf, and Pumilio). Strikingly, the motifs obtained from CCHC-zf domain proteins are mostly distinct, a notable exception being CPSF4 and RBBP6—both of which bind U-rich motifs and are involved in pre-mRNA cleavage and polyadenylation^42–44^. Altogether, this outcome indicates that sequence-specific RNA-binding is relatively common among CCHC-zfs.

### CLIP-seq data are consistent with lack of sequence specificity for ucRBPs

The RNAcompete pool we utilized here is designed to capture short, unstructured RNA-binding motifs. It is also capable of detecting RNA structure preferences^45^, but it was not designed to do so. We reasoned that the association of ucRBPs with cellular RNA might be explained by binding to long and/or structured motifs, which should be detected in cellular binding sites. To test this hypothesis, we analyzed eCLIP data published as part of ENCODE^46^. We curated a dataset of 31 eCLIP experiments (encompassing 26 proteins and two cell lines) that correspond to ucRBPs analyzed by RNAcompete (Supplementary Table S5). To these data, we applied PRIESSTESS^47^, a pipeline that produces models of RNA sequence and RNA structure binding specificity. We applied PRIESSTESS twice to each eCLIP experiment, once to identify short motifs (4–6) bases, and once to identify long motifs (7–12 bases) (see “Methods” for details).

For 12 of the 31 eCLIP experiments, no predictive motif models were produced by PRIESSTESS using either short or long motif settings due to a lack of enriched motifs in the eCLIP peaks. In contrast, 17 eCLIP experiments yielded similar motifs from both short and long settings, and the PRIESSTESS models containing either short or long motifs showed no overall difference in performance (P = 0.73; paired t-test) (Supplementary Fig. S2); indicating that long motifs are not prevalent. Strikingly, the motifs obtained for different proteins were often very similar to each other and contained little or no indication of preference for RNA structure (Supplementary Fig. S3).

For the remaining two ucRBPs, SLBP and NIP7, PRIESSTESS models were generated only with the long motif setting, and these models had good predictive capacity (area under the ROC curve = 0.68 on held-out data for both). In contrast to the models for the other ucRBPs, these models each contained long, structured motifs. The motifs in the PRIESSTESS SLBP model closely resemble the stem-loop sequence from which SLBP derives its name (Stem-Loop Binding Protein)^48^ (Supplementary Fig. S4A–C). The NIP7 motif closely resembles that of its interaction partner NHP2L1, which binds an internal loop sequence in the U4 snRNP^49^ (Supplementary Fig. S4D–F). Thus, even with relatively few peaks (SLBP-159, NIP7-293), this pipeline can detect larger structured motifs.

**Figure 5:**
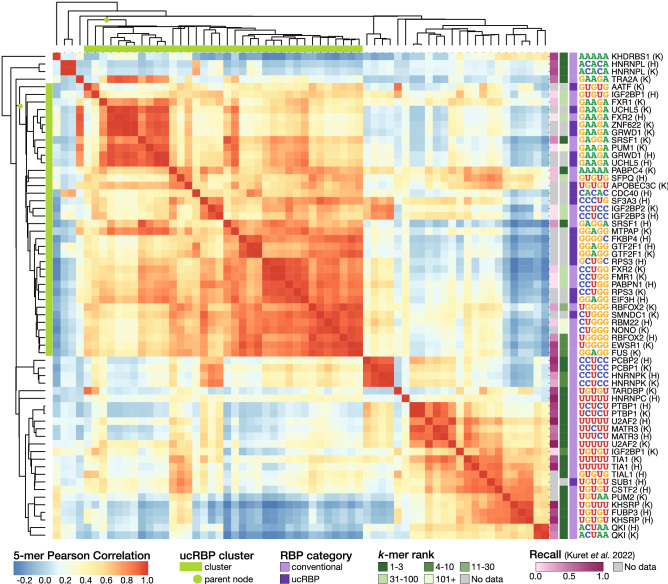
ucRBP eCLIP experiments are not enriched for unique motifs. Heatmap displays Pearson correlations of 5-mer frequencies between 64 eCLIP experiments. 5-mer frequencies were counted in the final set of merged peaks for each experiment as downloaded from ENCODE^46^. To the right of each row the assayed RBP and cell line (K—K562, H—HepG2) are displayed along with the most frequent 5-mer. RBP categories are indicated to the left of the most frequent 5-mer: conventional RBPs contain at least one RRM, KH, or PUF domain or have an in vitro derived motif, whereas ucRBPs are restricted to those that have been assayed by RNAcompete. For conventional RBPs, the first occurrence of the known in vitro derived IUPAC motif in rank ordered 5-mers (k-mer rank) is indicated. IUPAC motifs are available in Supplementary Table S6. Where corresponding RNA Bind-n-Seq (RBNS) data is available, the recall of the top eCLIP motif in the RBNS dataset, as calculated in Kuret et al.^72^ is shown. Higher values indicate better correspondence between eCLIP and RBNS experiments. Finally, at the top and left of the heatmap, the "ucRBP cluster" containing all but one of the ucRBPs is indicated with a green bar and the parent node of the cluster is highlighted. The heatmap was generated in R (version 4.1.3) using the pheatmap package (version 1.0.12).

To explore the surprising observation that many different ucRBPs yield short motifs that are related to each other we performed an all-by-all comparison of 5-mer frequencies, thus removing motif modeling as a variable. We also expanded the analyses to incorporate eCLIP experiments for 34 conventional RBPs (46 experiments) (Supplementary Table S6), for contrast. Clustering the matrix of Pearson correlations of 5-mer frequencies produced one major cluster that contained almost all ucRBPs, as well as numerous conventional RBPs (Fig. 5). Most proteins in this cluster fall into two sub-clusters: one composed of proteins that bind GAAGA-, GAGGA-, or GGAGG-like 5-mers, and one composed of proteins that bind other G-rich sequences. Among the well-studied conventional RBPs within this large cluster, the known binding specificity is typically not represented among the most frequent 5-mers (e.g. PUM1 which is known to bind UGUAHAUA is enriched for the GAAGA 5-mer, and PABPN1 which is known to bind poly(A) sequences is enriched for the CCUGG 5-mer^8^), suggesting that the sites captured by eCLIP are not dictated by the sequence specificity of the RBP.

In contrast, for most of the well-studied conventional RBPs outside of the main cluster, the most frequent 5-mers from eCLIP experiments almost uniformly display a close match to their known in vitro RNA-binding specificity, and form distinct clusters (e.g. HNRNPK, U2AF2, and QKI) (Fig. 5). These smaller clusters often correspond to the same protein analyzed in two different cell lines. One exception is the ucRBP SUB1, which yields a k-mer enrichment profile almost identical to that of CSTF2, a protein with which SUB1 physically associates^50^. CSTF2 is known to recognize GU-rich sequences downstream of the cleavage and polyadenylation (CPA) site^51^. In both SUB1 and CSTF2 eCLIP data, the top enriched 5-mer is GUGUG and the peaks for both proteins are predominantly found at CPA sites (median distance to CPA site: SUB1—0 bases, CSTF2—3 bases). These data suggest that the high similarity between SUB1 and CSTF2 likely result from their known association in cells and co-purification during eCLIP experiments.

### Most ucRBPs are abundant proteins

**Figure 6:**
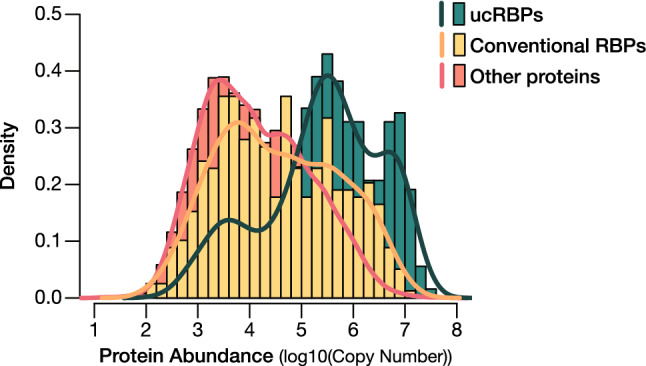
Protein abundance profiles of RBPs. Protein copy number estimates^52^ were cross-referenced with the ucRBPs analyzed in this study and conventional RBPs previously identified. Histograms show the distribution of protein abundance (log10 values of protein copy number) for ucRBPs analyzed in this study (green), conventional RBPs (yellow), and all other proteins (orange).

Finally, we sought to address why so many proteins associated with cellular RNA did not produce motifs in RNAcompete or eCLIP. Gross technical failure seems unlikely; the proteins analyzed by RNAcompete were produced and analyzed in parallel with canonical RBDs that had much higher success rates. We considered a variety of specific technical possibilities, but most could be excluded (see “Discussion”). The ucRBPs do, however, display an overall property that could readily explain their presence in interaction capture assays: ucRBPs are highly abundant in whole-cell mass spectrometry surveys and are often among proteins with the highest peptide counts^52^. Figure 6 shows that the range of abundance is markedly higher for ucRBPs relative to both conventional RBPs and all other proteins. Strikingly, of the top 10% most highly abundant proteins in HeLa cells^52^, 84% have been identified in one or more RNA interactome capture experiments^6^ (Supplementary Table S7, Supplementary Fig. S5A).

In addition, intrinsically disordered regions (IDRs), which have been associated with promiscuous interaction between proteins and RNA^53–55^, and are known to specifically mediate interactions between ucRBPs and RNA^11^, are enriched in the set of proteins captured by RNA interactome experiments (P = 3.0 × 10^–8^, 9.3% increase, Fisher’s Exact Test)^6^. Moreover, these proteins have significantly more amino acids in intrinsically disordered regions than proteins that are not captured (P = 2.6 × 10^–27^, 63.8% increase in mean; two-sided t-test) (Supplementary Table S8, Supplementary Fig. S5B). Coupled with high abundance, IDRs could partially explain the prevalence of sequence non-specific ucRBPs in RNA interactome capture.

## Discussion

We used RNAcompete to identify RNA-binding preferences for 23 sequence-specific ucRBPs. As RNA-binding is an inherent property of RBPs, identification of RNA-binding motifs for these proteins is an important first step in deciphering their function in RNA processing, metabolism, or post-transcriptional gene regulation. Among these newly discovered sequence-specific ucRBPs are many new and unusual cases. For example, ILF2, a known regulator of IL2, recognizes GC-rich RNA sequences, while two DNA-binding proteins, PURA and SSBP1, recognize a GA-rich RNA sequence and an RNA sequence with an AUG core, respectively. Approximately a third of the sequence-specific ucRBPs identified are ribosomal proteins, and several others have roles in human disease and development (e.g. PEG10, CNBP, NUDT16L1, PURA, SSBP1, and SERBP1)^29,34,56–63^. As such, the new motifs identified in this study could be used to characterize pathological mutations and/or the molecular determinants of RBP-RNA interactions. Surprisingly, RNAcompete-based analyses revealed specific and conserved RNA-binding activity for domains that normally have other functions (e.g. the hyaluronan binding domain, HABP4, in SERBP) in species that diverged hundreds of millions of years ago (i.e. human, zebrafish, and mouse), which supports the idea that the sequence specificity is of functional importance.

CCHC-zf proteins have roles in DNA-binding, protein–protein interactions, and are commonly associated with RNA-related processes^40,41,64–66^. The RNA-binding specificities for most CCHC-zf domains, if any, have not been previously determined, however. Nearly a third of CCHC-zf domains in this study displayed sequence specificity. Interestingly, motifs from the different CCHC-zfs analyzed are generally distinct, indicating flexibility in sequence preference, reminiscent of RRM, KH, and CCCH-zf domains (as well as C2H2-zf DNA-binding domains, where non-specific DNA-binding appears to facilitate rapid evolution of sequence specificity^67^). Moreover, as at least seven CCHC-zf proteins display sequence-specific RNA-binding, CCHC-zf now represents the fourth largest class of sequence-specific RBDs in human (behind RRM, KH, and CCCH-zf). Taken together, these data suggest that inclusion of the CCHC-zf domain family among the canonical sequence-specific RBDs would be reasonable and appropriate.

A striking observation from this study is that the vast majority of ucRBPs identified through RNA interactome capture, whether analyzed by RNAcompete or eCLIP, did not display RNA sequence specificity. Technical reasons for failure in RNAcompete experiments include aberrant protein production, and possible shortcomings of the RNAcompete assay itself (e.g. the inability to detect complex motifs or RNA secondary structure). For the former, the proteins examined were affinity-purified and therefore soluble, consistent with proper folding. For the latter, RNAcompete is effective in capturing small RNA bipartite motifs for proteins such as hnRNPL and hnRNPLL^68^ as well as components of larger RNA sequences such as the CNGGN hairpin-pentaloop consensus site for Vts1^36,69^ and the GGAG consensus partial binding site contained in let-7 pre-miRNA^70,71^. Additionally, binding to larger G-quadraplexes, as described for CNBP^30^, could be detected as short primary sequence motifs and indeed, the CNBP motif we obtained resembles the potential CNBP-bound G-quadraplexes described in Ref.^30^.

The ucRBPs could conceivably bind only to very long and/or completely structured sites, but we did not detect such sites in eCLIP data for the vast majority of ucRBPs, instead finding either no sequence specificity or sequences that are frequently shared across many unrelated experiments. In a separate study, Kuret et al.^72^ used a very different strategy to analyze all ENCODE eCLIP data, but nonetheless made similar findings, including a large cluster of unrelated RBPs that crosslink to G-rich sequences. These sequences were proposed to represent common contaminants in eCLIP data. Thus, analysis of eCLIP data appears to confirm RNAcompete results for many RBPs.

It is thus unclear whether the observed lack of sequence-specific RNA-binding is an inherent property of ucRBPs (i.e. they bind RNA, but non-specifically), or is a consequence of other confounding factors such as transient RNA-binding activity in cells, high protein abundance, and/or technical issues with RNA interactome capture experiments. DNA and rRNA contamination were common in early RNA interactome capture studies, suggesting a potential for false identification of DNA-binding or structural ribosomal proteins as *bona fide* mRNA-binding RBPs^73,74^. In “enhanced” RNA interactome capture experiments^74^, DNA and 25S RNA contamination issues have been largely circumvented. 18S rRNA contamination remains, however, albeit at significantly reduced levels^74^. Given that many of the ucRBPs have known RNA-related functions, it is also conceivable that they interact with RNA via mechanisms that do not rely on intrinsic sequence specificity (e.g. recruitment). Indeed, for SUB1 and NIP7, cellular RNA associations seem to be mediated by interactions with CSTF2 and NHP2L1, respectively. Additionally, proteins identified through RNA interactome capture studies can crosslink to RNA due to non-specific RNA-binding or transient associations^15,75^. Analogous features have been observed for chromatin proteins, which are distinguished from transcription factors by their lack of DNA sequence specificity, but nonetheless crosslink effectively to cellular DNA in ChIP-seq experiments^76,77^.

Finally, we propose that greater precision in terminology would be beneficial. “RNA-binding protein” should be used only to describe proteins that bind RNA with high sequence or structure specificity, whereas “nonspecific RNA-binding protein (nsRBP)” should be used to describe proteins that bind RNA non-specifically, and “RNA-associated protein” would describe proteins that associate with RNA in cells but do not possess intrinsic RNA-binding activity. Different terms are already used for equivalent types of DNA-associated proteins: “transcription factors”, “low specificity DNA-binding proteins”, and “chromatin proteins”. We propose that, at the very least, the class of “all proteins that contact RNA in cells” should not be conflated with the (apparently much smaller) sequence-specific subset.

## Methods

### RNAcompete

The RNA pool generation, RNAcompete pulldown assays, and microarray hybridizations were performed as previously described^12,36,71^. Briefly, RNAcompete experiments employed defined RNA pools that are generated from 244 K Agilent custom DNA microarrays. The RNA pool is designed using a single de Bruijn sequence^71,78^ of order 11 that was subsequently modified to minimize secondary structure in the designed sequences and minimize intramolecular RNA cross-hybridization. After these modifications, not every 11-mer is represented but each 9-mer is represented at least 16 times. To facilitate internal data comparisons, the pool is split computationally into two sets: Set A and Set B. Each set contains at least 155 copies of all 7-mers except GCTCTTC and CGAGAAG, which are removed because they correspond to the SapI/BspQI restriction site used during DNA template pool generation. A φ2.5 bacteriophage T7 promoter initiating with an AGA or AGG sequence is added at the beginning of each probe sequence in the DNA template pool to enable RNA synthesis. The final RNA pool consists of 241,399 individual sequences up to 41 nucleotides in length. The microarray design can be ordered from Agilent Technologies using AMADID# 024519. During the pulldown component of RNAcompete assays, 20 pmol of full-length GST-tagged ucRBPs and RNA pool (1.5 nmoles) are incubated in 1 mL of Binding Buffer (20 mM HEPES pH 7.8, 80 mM KCl, 20 mM NaCl, 10% glycerol, 2 mM DTT, 0.1 μg/μL BSA) containing 20 μL glutathione Sepharose 4B beads (Cat #17-0756-05, GE Healthcare; pre-washed 3 times in Binding Buffer) for 30 min at 4 °C, and subsequently washed four times for two minutes with Binding Buffer at 4 °C. The RNA is then recovered by thermal elution and labeled with Cy3 or Cy5 using the Kreatech ULS Labeling Kit. The labeled RNA is denatured and hybridized to a fresh single-stranded Agilent array of the same design, using a Tecan HS4800 Pro Hybridization Workstation. Samples are hybridized for 20 h at 42 °C, washed, and scanned. Images are processed using Imagene software version 8.0, with manual spot flagging.

### RNAcompete data processing

Normalization of microarray probe intensities, calculation of 7-mer Z-scores, and derivation of motifs were performed as described in^12,36,71^. In this study, however, logos were generated from PFMs using ggseqlogo^79^.

### ucRBP constructs

Full-length (for genome-wide analysis) or truncated (for domain analysis) ucRBP coding sequences were cloned into the AscI and SbfI restriction sites in a modified pDEST-Magic vector (pTH6838)^71^, resulting in an expression construct N-terminally-tagged with GST. The vector map and sequence for pTH6838 can be found at http://hugheslab.ccbr.utoronto.ca/supplementary-data/RNAcompete_eukarya/. Constructs were either commercially synthesized by BioBasic or cloned “in-house” using the Superscript II One-Step RT-PCR System (Cat #10928042, Invitrogen, following the manufacturer’s recommendations), FirstChoice Human Total RNA Survey Panel (AM6000, Ambion) as template, and gene-specific primers. For analysis of RBDs, up to 50 amino acids of flanking sequence was included (less if the end of the polypeptide or a neighboring domain is encountered). Construct sequences are provided in Supplementary Table S1.

### Protein purification

GST-tagged ucRBP expression constructs were transformed into *Escherichia coli* C41 cells (Lucigen), and protein expression was induced by adding IPTG (1 mM final) to log phase cell cultures and incubating overnight at 16 °C. Supplementary Table S1 provides information on proteins. Cell lysates were prepared by sonication, and then added to GST resin (Cat #17-5279-01, GE Healthcare) for binding. After washing to remove non-specific binders, GST-tagged proteins were eluted using 250 mM NaCl, 50 mM Tris–HCl (pH 8.8), 30 mM reduced glutathione, 10 mM BME, and 20% Glycerol. Protein concentration and purity were estimated by SDS-PAGE and Bradford assay.

### RNAcompete pass/fail classifier

Training and testing data for our classifier were generated by manually annotating 471 prior RNAcompete experiments for proteins containing RRM, KH, CCCH-zf, or SAM domains as passed or failed experiments (Sasse et al., in preparation). Each experiment was annotated as a “Pass” if it showed an obvious visible correlation in k-mer enrichment between the Set A and Set B probes, the two sets produced visibly similar motifs, and the motif was not composed of k-mers that are found in many unrelated experiments (e.g. simple repeat sequences). Similar quality control steps used in RNAcompete microarray data analysis have been outlined in more detail elsewhere^12^. We annotated the rest of the experiments as “Fails”, resulting in 229 passes and 242 fails. Forty of these experiments (20 passes and 20 fails) were held out for testing, the majority of which were performed on RBPs with well-described motifs. The remainder were used to train the classifier (Supplementary Table S2).

As features for the classifier, we used various statistics generated from the 7-mer Z-scores for the Set A and Set B probes. These features were: the correlation in 7-mer Z-scores between Set A and Set B probes, the overlap in the top ten 7-mers between the two sets, the individual Z-scores for the top ten 7-mers in each set, the skewness and kurtosis of the two Z-score distributions, and the highest 7-mer Z-score from the merged sets. Features capturing the presence of 26 known RNAcompete artifacts (k-mers of lengths 4–7) were also used: the number of top ten Set A and Set B 7-mers containing each of the artifacts were used as individual features, along with the combined sum of all the artifact counts. Finally, features capturing information about the Set A and Set B motifs were added: the information content of each motif and the similarity between the two motifs as calculated by TOMTOM^80^ (Supplementary Table S2).

We trained a logistic regression (LR) model using the LogisticRegression function from scikit-learn^81^ with BayesSearchCV from scikit-optimize (https://scikit-optimize.github.io) to determine the optimal L1 (i.e., LASSO) regularization strength. This resulted in a classifier with nearly perfect performance on the held-out test data (AUROC = 0.99). The LR probability estimate for passed RNAcompete experiments in the held-out set ranged from 0.43 to 1.00 (mean = 0.92) and for failed experiments from 7.8 × 10^–5^ to 0.47 (mean = 6.1 × 10^–2^) (Supplementary Fig. S1A, Supplementary Table S2).

We applied the classifier to all ucRBP experiments, thresholding the results such that experiments with an LR probability estimate ≤ 0.35 were determined to have failed, experiments with an LR probability estimate ≥ 0.65 were determined to have passed, and experiments that fell between were manually checked (Supplementary Fig. S1B, Supplementary Table S3).

Of the 20 experiments that required manual checking, 17 were experiments on full-length ucRBPs and three were experiments using truncated constructs. Based on duplicate experiments, the similarity of the motif to artifacts, and the similarity of the motif to motifs for homologous proteins, each was determined to have passed or failed. Specific reasoning for each experiment is detailed in Supplementary Table S3.

### Domain alignments

To generate the alignments in Figs. 3 and 4, we first performed multiple sequence alignment on the amino acid sequences of the domains, or domain-containing regions, using Clustal Omega^82^ for each of the six ucRBDs examined. Domain sequences were input to COBALT^83^ for visualization using the “Show Differences” colouring setting. HABP4, Nudix hydrolase, and L7Ae domain-containing proteins each harbored only a single copy of the domain, so the alignments were anchored on the representative protein domain to display detailed differences in the amino acid sequences. Due to the presence of multiple domain occurrences in some proteins containing TPR, CCHC-zf, and RanBP2-zf ucRBDs, alignments were not anchored in order to show the full length of all domain-containing regions. Details on the domain sequences are found in Supplementary Table S4.

### eCLIP data

Merged peak BED files were downloaded for all eCLIP experiments in the ENCODE data portal^46^. We compiled a set of 31 experiments (26 unique proteins) that were performed on proteins in our ucRBP set. This set of experiments was used for the PRIESSTESS^47^ analysis (Supplementary Table S5). For the eCLIP experiment 5-mer frequency comparisons, we reduced this set to experiments that contained at least 1000 peaks to reduce noise, resulting in 18 experiments (14 proteins). We also curated a set of conventional eCLIP experiments by collecting experiments performed on proteins that both have published in vitro data available (RNA Bind-n-Seq (RBNS) or RNAcompete) and contain an RRM, KH, or PUF domain. The conventional RBP eCLIP set was also reduced to experiments that contain at least 1000 peaks, resulting in 46 experiments encompassing 34 proteins. Experiment details can be found in Supplementary Table S6.

To prepare ucRBP eCLIP data for PRIESSTESS, each peak was extended by 20 bases upstream to ensure the full binding site was included, and negative sets were generated by taking sequences of the same size as each peak from 300 bases upstream. Before passing the sequences to PRIESSTESS, 50 flanking bases were added up- and down-stream in addition to the upstream 20 base extension. These 50 flanking bases were added to provide context for RNA folding and are removed prior to motif identification and later steps; only the additional 20 upstream bases remain, as these constitute part of the binding site. We ran PRIESSTESS twice for each eCLIP experiment, once with default settings (motif size 4–6), and once with the motif size set to 7–12 (-minw 7-maxw 12). Further increasing motif length (13–20) in PRIESSTESS runs resulted in either no enriched motifs being identified or a model with worse predictive power for all experiments. Due to the small number of sequences in many of the experiments, the p-value threshold for significantly-enriched motifs identified by STREME was increased to 0.1. Note that while this increases the number of motifs used in the logistic regression step of PRIESSTESS, it will not lead to the creation of predictive models if the motifs are not representative of the binding specificity; either the LASSO regularization will set all motif weights to zero, or the final model will fail to identify bound sites in the held-out data. AUROC values on held-out data output by PRIESSTESS were compared (short motif model vs. long motif model) using a paired t-test.

To compare k-mer similarity across ucRBP and conventional RBP eCLIP experiments, 5-mers were counted in peak sequences for each eCLIP experiment. Pearson correlations between 5-mer counts for each pair of experiments were calculated and experiments were clustered using hierarchical agglomerative clustering with centroid linkage. To identify the k-mer rank of the known in vitro motif, we curated IUPAC motifs from CisBP-RNA^71^ and RBNS motifs^84^, except in the case of CSTF2, for which the motif is known to be a GU-rich sequence^85^. Curated IUPAC motifs can be found in Supplementary Table S6. For each experiment, 5-mers were ranked based on frequency and the first occurrence of the IUPAC motif was identified. Recall values shown in Fig. 5 were downloaded from Kuret et al*.*^72^ additional file 7.

### Protein abundance

We used data from mass spectrometric analysis of endogenously expressed proteins in HeLa cells (Supplementary Table 3 from^52^) to survey the relative abundance of ucRBPs. Here, histograms corresponding to log10 values for protein copy number were plotted for ucRBPs, conventional RBPs and all “other” proteins identified (Supplementary Table S7). ucRBPs and conventional RBPs were compiled from this study and RBPDB^8^, respectively.

### Intrinsically disordered regions

To analyze the prevalence of IDRs in the RNA interacting proteome, we collected IDR data from MobiDB^86^, specifically the number of amino acids in each protein that are within an IDR as determined by MobiDB-lite^87^. We reduced the set of proteins to those in the UniProt human proteome (UP000005640) that have been reviewed. Each of the proteins was then annotated as belonging to (or not belonging to) the set of proteins identified in interactome capture experiments as curated on RBPbase^6^ (Supplementary Table S8).

## Supplementary Information

Supplementary Figures.

Supplementary Table S1.

Supplementary Table S2.

Supplementary Table S3.

Supplementary Table S4.

Supplementary Table S5.

Supplementary Table S6.

Supplementary Table S7.

Supplementary Table S8.

## Data Availability

RNAcompete data have been deposited at GEO (GSE215198). Data underlying figures in the manuscript, as well as motifs for positive results, are housed at http://datah.ccbr.utoronto.ca/ucRBP. Code for RNAcompete probe normalization and motif generation is housed at https://github.com/morrislab/RNAcompete. The script and data to recreate the RNAcompete experiment classifier can be found at https://github.com/morrislab/RNAcompete_classifier.
