# Supplementary material for: RNA-binding proteins that lack canonical RNA-binding domains are rarely sequence-specific
Source: Sci Rep. 2023 Mar 31;13:5238. doi: 10.1038/s41598-023-32245-9 (PMC10066285; doi:10.1038/s41598-023-32245-9)
Supplement: Supplementary file 1 — Supplementary Figures. [file 41598_2023_32245_MOESM1_ESM.pdf]

RNA-binding proteins that lack canonical RNA-binding domains are rarely sequence-specific

Debashish Ray[1,#], Kaitlin U. Laverty[1,2,#], Arttu Jolma[1], Kate Nie[1,2], Reuben Samson[1,2], Sara E. Pour[1,2], Cyrus L. Tam[4], Niklas von Krosigk[1,2], Syed Nabeel-Shah[1,2], Mihai Albu[1], Hong Zheng[1], Gabrielle Perron[3], Hyunmin Lee[1], Hamed Najafabadi[3], Benjamin Blencowe[1,2], Jack Greenblatt[1,2], Quaid Morris[1,2,4,*], Timothy Hughes[1,2,*]

[1] Donnelly Centre, University of Toronto, Toronto, ON M5S 3E1 Canada
[2] Department of Molecular Genetics, University of Toronto, Toronto, ON M5S 1A8 Canada
[3] Department of Human Genetics, McGill University, Montréal, QC H3A 0G1 Canada, and McGill Genome Centre, Montréal, QC H3A 0G1, Canada
[4] Computational and Systems Biology Program, Memorial Sloan Kettering Cancer Center, New York, NY, USA Tri-institutional Training Program in Computational Biology and Medicine, Weill Cornell Medicine, New York, NY, USA

[#] Co-first authors
* To whom correspondence should be addressed: t.hughes@utoronto.ca; morrisq@mskcc.org

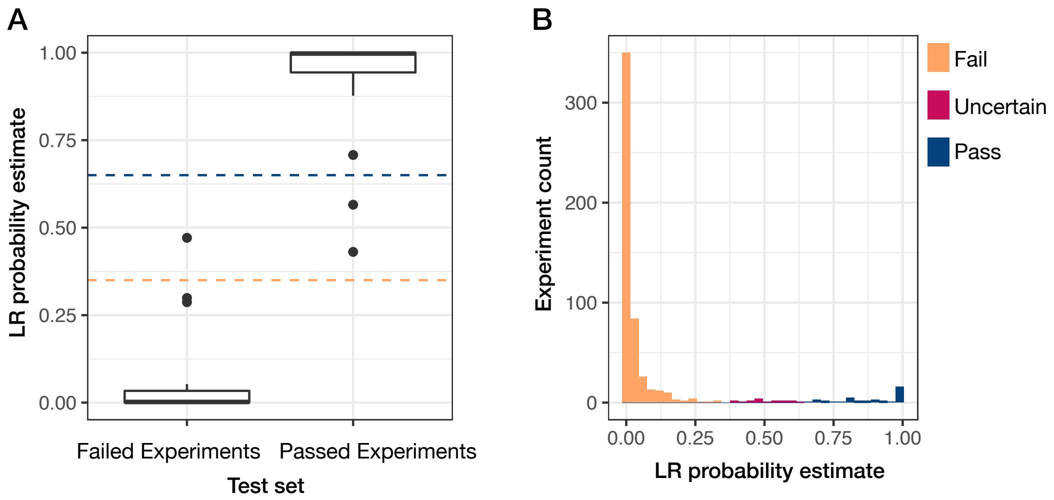

Supplementary Figure S1. RNAcompete pass/fail classifier results. (**A**) The output of the logistic regression (LR) classifier trained to differentiate passed vs. failed RNAcompete experiments is shown on the 40 experiment test set (i.e., held-out data) (see **Supplementary Table S2**). Thresholds are drawn at 0.65 (dashed blue line) and 0.35 (dashed orange line) to indicate the range (0.35 – 0.65) in which the classifier output is ambiguous ("uncertain"). (**B**) RNAcompete pass/fail classifier results for the 558 experiments (492 individual proteins) performed on full-length ucRBP constructs. Bars are coloured to differentiate between three ranges: 0 – 0.35, failed experiments; 0.35 – 0.65, "uncertain" experiments that require manual checking; and 0.65 – 1, passed experiments.

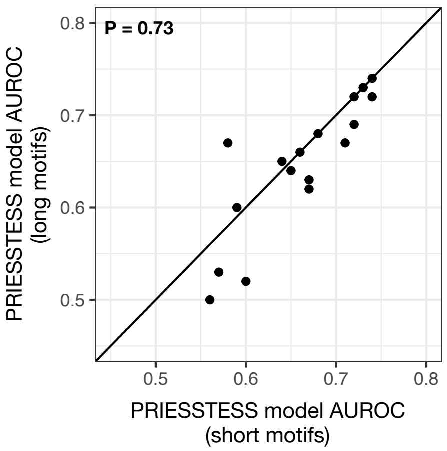

Supplementary Figure S2. Comparison of PRIESSTESS models trained with short and long motifs. PRIESSTESS[6] models were trained for all eCLIP experiments corresponding to ucRBPs in this study (**Supplementary Table S7**). PRIESSTESS was run twice, once with the motif length set to 4-6 (short motifs) and once with the motif length set to 7-12 (long motifs). For twelve experiments, no predictive models were generated in either run - this was due to a lack of enriched motifs or the resulting model(s) showed poor ability to identify bound sites in held-out data (AUROC ≤ 0.55). For 17 experiments, both a long and short motif model were produced, at least one of which had an AUROC > 0.55. Here, a comparison of performance on held-out data (as AUROC) between short and long motif models for the same eCLIP experiment is displayed. Neither set of models significantly outperforms the other (P = 0.73; paired t-test). Note that SLBP and NIP7 are not included in this plot as a model was produced only with the long motif setting.

RBP (cell line)	PRIESSTESS model (AUROC)	RNAcompete motif(s)	eCLIP 5-mer
UCHL5 (K562)	2-letter (0.72)	RNCMPT01276	GAAGA
ZNF622 (K562)	(0.74)	RNCMPT01808	GAAGA
GRWD1 (K562)	(0.69)	RNCMPT01849	GAAGA
GRWD1 (HepG2)	(0.73)	RNCMPT01849	GAAGA
UCHL5 (HepG2)	2-letter (0.66)	RNCMPT01276	GAAGA
SF3A3 (HepG2)	(0.63)	RNCMPT00640 RNCMPT00900	CCCUG
MTPAP (K562)	(0.62)	RNCMPT00647	GGAGG
FKBP4 (HepG2)	(0.68)	RNCMPT01839	GGGGC
GTF2F1 (HepG2)	(0.67)	RNCMPT01850	GGAGG
GTF2F1 (K562)	2-letter (0.72)	RNCMPT01850	GGAGG
RPS3 (HepG2)	(0.65)	RNCMPT01742	GCUGC
RPS3 (K562)	4-letter (0.67)	RNCMPT01742	CCUGG
EIF3H (HepG2)	(0.60)	RNCMPT01213	GGAGG
SUB1 (HepG2)	(0.64)	RNCMPT00369 RNCMPT00561	UGUGU
NIP7 (HepG2)*	7-letter (0.68)	RNCMPT01700	UGAUG
SLBP (K562)*	7-letter 2-letter 2-letter (0.68)	RNCMPT01759	AAGGC

* eCLIP experiment has fewer than 1000 peaks and therefore is not in **Figure 5**

RNA structure alphabets

2-letter alphabet
P - Paired
U - Unpaired

4-letter alphabet
P - Paired
U - External
L - Hairpin loop
M - Other loop type

7-letter alphabet
L - Left paired
R - Right paired
E - External
H - Hairpin loop
B - Bulge
M - Multi-loop
T - Internal loop

Proportion of total PRIESSTESS model weight

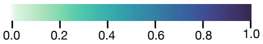

Probability of successful experiment
(Classifier result)

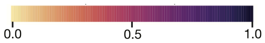

Supplementary Figure S3. eCLIP ucRBP PRIESSTESS models. Predictive PRIESSTESS models using motifs of length 7-12 were successfully generated for 16 eCLIP experiments and are displayed in the table above. The ucRBP experiments excluded from this figure either produced no model due to a lack of enriched motifs or the resulting model(s) showed poor ability to identify bound sites in the held-out data (AUROC ≤ 0.55). The name of the RBPs and cell lines used in eCLIP experiments are specified in the first column. The top motifs retained in the PRIESSTESS model, up to a maximum of three, are displayed in the second column. PRIESSTESS motifs are shown in descending order based on their contribution to the model weight; a higher model weight indicates the motif has a greater impact in defining the RNA-binding specificity of an RBP. Additionally, these motifs are comprised of two parts, sequence at the top (if included) and structure at the bottom (if included). Structural alphabets are indicated below motifs containing structure, and the letter representations of RNA structural elements are defined to the right of the table. The AUROC on held-out data is displayed in parentheses beside the model. For comparison, RNAcompete motif(s) for the ucRBP along with the probability that the RNAcompete experiment was successful according to the RNAcompete pass/fail classifier is shown in the third column, and the most frequent 5-mer in the eCLIP peaks is shown in the fourth column. Experiments are ordered based on their order of appearance in **Figure 5**. The last two RBPs (NIP7 and SLBP), highlighted with asterisks, were not included in **Figure 5** as fewer than 1000 peaks were available.

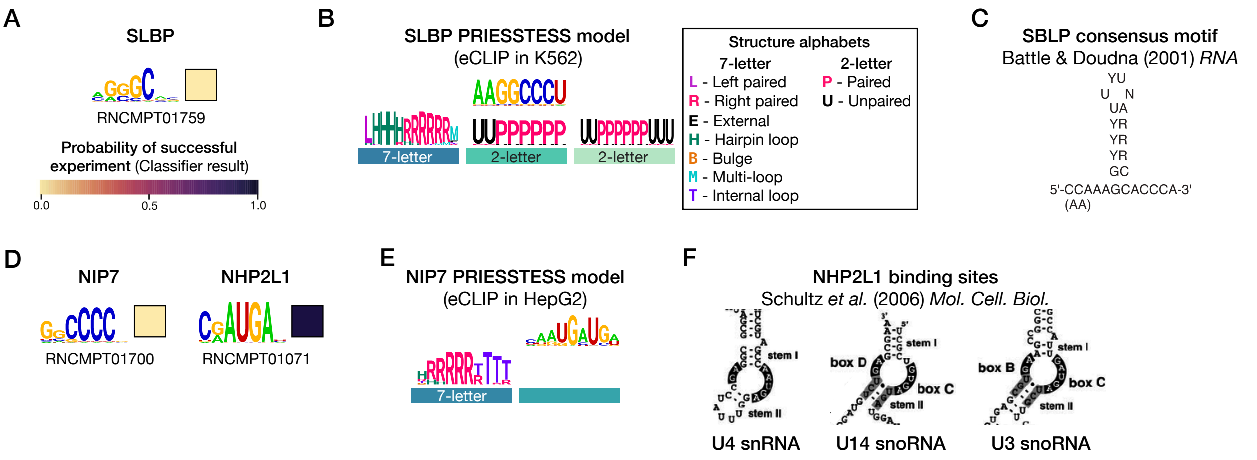

Supplementary Figure S4. SLBP and NIP7 eCLIP experiments reveal structured binding motifs. (**A**) The RNAcompete motif identified for human SLBP and the probability that the RNAcompete experiment was successful according to the RNAcompete pass/fail classifier. (**B**) PRIESSTESS model trained using eCLIP data for SLBP. All motifs retained in the final PRIESSTESS model are displayed in descending order based on their contribution to the model weight; a higher model weight indicates the motif has a greater impact in defining the RNA-binding specificity of the RBP. Motifs are comprised of two parts, sequence at the top (if included) and structure at the bottom (if included). Structural alphabets are indicated below motifs containing structure. (**C**) The SLBP consensus binding site as defined by Battle and Doudna[4] (the image is cropped from Figure 1B of their paper). (**D**) RNAcompete motifs and RNAcompete pass/fail classifier results for NIP7 and NHP2L1 motifs. (**E**) PRIESSTESS model trained using eCLIP data for NIP7. (**F**) Three experimentally identified binding sites of human NHP2L1 (also known as SNU13 and 15.5K) as shown in Schultz *et al.*[5] (binding site images are cropped from Figure 1 of their paper).

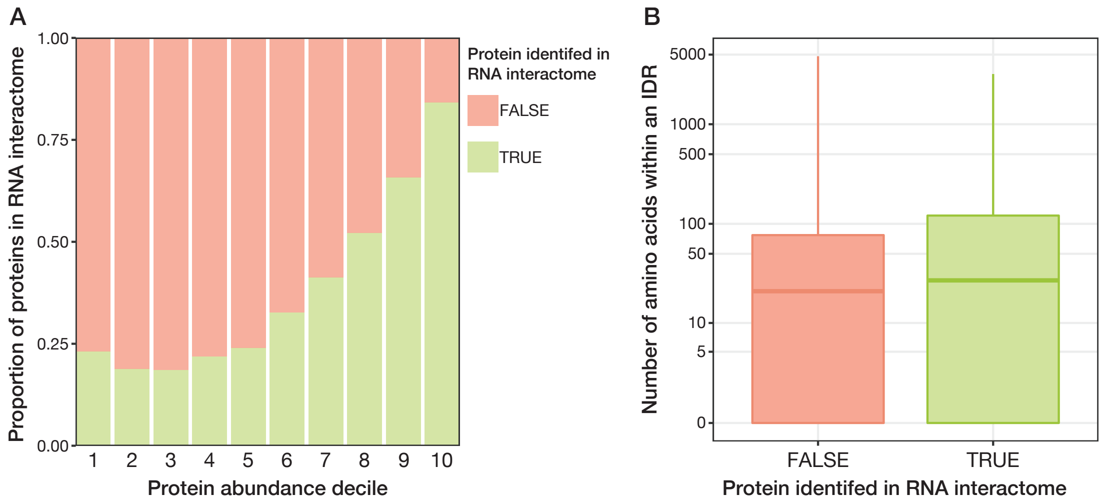

Supplementary Figure S5. Characteristics of proteins found in the RNA interactome. Comparison of the 4257 human RBPs as curated in RBPbase[1] to the rest of the human proteome. (**A**) Proteins are split into deciles based on abundance in HeLa cells[2], with the 10th decile containing the top 10% most abundant proteins. The proportion of proteins within each decile found in the RNA interactome is displayed: RBPs identified through RNA interactome capture studies are represented by a light green bar (TRUE) and the remaining proteins from the human proteome are represented by a pink bar (FALSE). (**B**) The number of amino acids within an IDR (as calculated by MobiDB-lite[3]) for all reviewed proteins in the UniProt human proteome. Proteins identified in RNA interactomes (light green; TRUE) have a significantly higher number of amino acids within IDRs than the remaining proteins from the human proteome (pink; FALSE) (P = 2.56E-27, 63.8% increase in mean; unpaired t-test).

References

1 Gebauer, F., Schwarzl, T., Valcarcel, J. & Hentze, M. W. RNA-binding proteins in human genetic disease. *Nat Rev Genet* **22**, 185-198, doi:10.1038/s41576-020-00302-y (2021).

2 Bekker-Jensen, D. B. *et al.* An Optimized Shotgun Strategy for the Rapid Generation of Comprehensive Human Proteomes. *Cell Syst* **4**, 587-599 e584, doi:10.1016/j.cels.2017.05.009 (2017).

3 Necci, M., Piovesan, D., Clementel, D., Dosztanyi, Z. & Tosatto, S. C. E. MobiDB-lite 3.0: fast consensus annotation of intrinsic disorder flavours in proteins. *Bioinformatics*, doi:10.1093/bioinformatics/btaa1045 (2020).

4 Battle, D. J. & Doudna, J. A. The stem-loop binding protein forms a highly stable and specific complex with the 3' stem-loop of histone mRNAs. *RNA* **7**, 123-132, doi:10.1017/s1355838201001820 (2001).

5 Schultz, A., Nottrott, S., Watkins, N. J. & Luhrmann, R. Protein-protein and protein-RNA contacts both contribute to the 15.5K-mediated assembly of the U4/U6 snRNP and the box C/D snoRNPs. *Mol Cell Biol* **26**, 5146-5154, doi:10.1128/MCB.02374-05 (2006).

6 Laverty, K. U. *et al.* PRIESSTESS: interpretable, high-performing models of the sequence and structure preferences of RNA-binding proteins. *Nucleic Acids Res* **50**, e111, doi:10.1093/nar/gkac694 (2022).
